# Molecular characterization of a porcine teschovirus HuN-1 isolate proliferating in PK-15 cell

**DOI:** 10.1186/s12917-018-1456-6

**Published:** 2018-04-27

**Authors:** Molin Chen, Wei Tang, Xiuguo Hua

**Affiliations:** 1 Shanghai Key Laboratory of Veterinary Biotechnology, School of Agriculture and Biology, Shanghai Jiao Tong University, Shanghai, 200240 China; 2 Department of Library, Hunan University of Science and Engineering, Yongzhou, 425199 People’s Republic of China

**Keywords:** Swine, Teschovirus, Complete genome, PK-15 cell

## Abstract

**Background:**

Porcine teschoviruses (PTVs) are small non-enveloped viruses with single-stranded, positive sense genomic RNA, belonging to the family *Picornaviridae*. Natural infections of teschoviruses are limited to pigs.

**Results:**

In this study, a PTV HuN-1 was found that it could be proliferated in PK-15 cell, and it came from the pig fecal samples from Hunan province, in central China. The complete genome of the HuN-1 was amplified by RT-PCR and sequenced. The complete genome of HuN-1 isolate is 7098 nt, which shares the highest sequence identity (85.9%) with the PTV 8 strain of Jilin/2003/2 and Fuyu/2009/2. The HuN-1 isolate contains only one ORF (from 320 to 7039 nt) coding a 2240 amino acid polyprotein. Aligned sequences show that more mutations occurred in the structural region than in the nonstructural region. Phylogenetic analysis showed that HuN-1 isolate did not clustered with the hitherto reported strains, according to P1 sequences, forming a subgroup in the PTV cluster.

**Conclusion:**

In this study, complete genome of PTV HuN-1 was cloned and sequenced. Detection and characterization of further PTV strains from different geographic areas are important to understand the worldwide distribution and heterogeneity (serotype) of PTVs and their association with symptomatic infections in pigs.

## Background

Porcine teschoviruses (PTVs) are small non-enveloped viruses with single-stranded, positive sense genomic RNA, belonging to the family *Picornaviridae*. Because of the nucleotide acid stability of the PTV, it was considered originally as porcine enteroviruses (PEVs) of cytopathogenic effect (CPE) group I, but has been classified as a separate genus, including former PEVs 1–7 and 11–13. Now, PTVs comprise at least thirteen serotypes identified [1–6]. Currently, PTV infections are diagnosed by RT-PCR [5–8] which replaced virus isolation in cell cultures and differentiation of serotypes by serological assays [9, 10].

Natural infections of teschoviruses are limited to pigs [11]. PTVs are the causative agents of severe and mild neurological disorders known as Teschen/Talfan disease, reproductive failure, pneumonia, and dermal lesions of swine [12–16]. Marked differences in neurovirulence among various strains of each serotype have been reported [17].

The PTV genome is 7.0–7.2 thousand nucleotides in length, and encodes a single polyprotein. The teschoviruses have the same genome organization: 5′-UTR, leader (L) protein, four structural proteins (VP4, VP2, VP3, and VP1), seven non-structural proteins (2A-2C, 3A-3D) and 3′-UTR [3, 18].

In this study, the complete nucleotide sequences and detailed genomic organization of PTV HuN-1 isolate are presented, and an analysis of the complete nucleotide sequences is reported. The existence of diversity and genetic recombination in porcine teschovirus are further confirmed.

## Methods

### Propagation and isolation of PTVs

Propagation and isolation of PTVs were performed following procedures. Briefly, PK-15 cells in our lab were maintained in Dulbecco’s modified Eagle’s medium (DMEM), and 10% (*v*/v) fetal bovine serum. Fecal sample of PTV positive is filtrated by 0.22 μm filter. Cell monolayers (25 cm^2^ flasks) were covered with the Filtrate for adsorption of one hour. And then, discuss the filtrate and add fresh media with 2% fetal bovine serum for viral propagation at 37 °C, 5% CO_2_. Observing the cytopathic effect (CPE), incubation was ended when 90% of the monolayers were destroyed. Blind passages three time. Then for ten generations. The sample of PTVs was isolated and purified by the plaque three times. After three freeze–thaw cycles, the specimen was clarified prior to storage at − 80 °C until used. Collection of pig fecal samples was performed after obtaining consent from the pig farm. All study protocols were approved by the Institutional animal care and use committee of the Shanghai Jiao Tong University. Experimental animals license Number: SYXK (shanghai) 2013–0052.

### RT-PCR detection of PTVs

RNA was extracted from 450 μl of the specimen with TRIzol Reagent (Invitrogen, USA), following the manufacturer’s instructions. The isolated RNA was dissolved in 25 μl RNase-free dH_2_O. cDNA synthesis was performed using a PrimeScript_RT Reagent Kit (Takara, Dalian, China). Briefly, purified viral RNA 16 μl was mixed with 4 μl of PrimeScript_RT Master Mix to a total volume of 20 μl. The reaction mixture was heated to 42 °C for 5 min, chilled on ice and then incubated for 1.5 h at 37 °C followed by 5 s at 85 °C to inactivate the reverse transcriptase. According to the reference [6], a pairs of primers were synthesized to detect PTVs based on the conserved sequences in 5’-UTR region (primer F: 5′-GTGGCGACAGGGTACAGAAGAC-3′; primer R: 5′-GGCCAGCCGCGACCCTGTCAG-3′).

### PCR amplification of teschovirus HuN-1 genome

The primers were designed with Primer 5.0 and synthesized by Life Technologies of Shanghai, China. According to the reference genomic sequences of porcine teschoviruses from GenBank, Eight pairs of primers (as Table 1 showed) was designed to amplify the teschovirus HuN-1 isolate genome.

**Table 1:** Primers were designed to amplify the Full-length genome of PTV HuN-1

Primers	Nucleotide Sequences	Position	Length(nt)
P1-F	5′-CTCCCTTTGAATTTGTAA-3′	1–18	382
P1-R	5′-GGCCAGCCGCGACCCTGTCAG-3′	362–382	
P2-F	5′-GGACTGGACTTGTGCTGCC-3′	344–362	1328
P2-R	5′-ATRTCAACRCTRGGTGTTCCTCC-3′	1648–1671	
P3-F	5′-CCCTAGGACAAATTCMTCAGCAA-3′	1522–1544	978
P3-R	5′-CCTGTTTCTGCAGGTTGMAGGGG-3′	2477–2499	
P4-F	5′-GGTCACGGGGATACATCACTA-3′	2316–2336	988
P4-R	5′- AACAGGGAGAAGTTTGTAGCA −3′	3262–3282	
P5-F	5′-ATCTTATTCAAATGAATCTAC-3′	3172–3192	797
P5-R	5′-YTCTGTCTGGRTGCCTGTAATT-3′	3947–3968	
P6-F	5′- CGGCTCAAAACCTGGAGAACT −3′	3839–3859	789
P6-R	5′- TAGGGTATGTTTGCCATGTTTA −3	4606–4627	
P7-F	5′-AAGCCTGACGGGACACTTGAT-3	4493–4513	1457
P7-R	5′-GGTTCAAGAAAGTCTGGTGGC-3	5929–5949	
P8-F	5′- GCCAACATTTGTGTGTGGTGATC -3	5743–5765	1356
P8-R	5′-AACTAAAACTACTCAAGCAACAGGC-3	7074–7098	

Reference strains were used to design the primers are as follows: CH/IMH/03 (DQ355222); JF613 (GU446660); 10BJ02 (JQ975417); HB-2010 (JQ664746); Jilin/2003/2 (JN710381); NC_003985

PCR was carried out using 2 μl of cDNA and a master mix containing 5 μl 10х LA PCR buffer, 2 μl of each primer (20 μM), 8 μl dNTP mixture (2.5 mM each), 0.5 μl LA Taq (Takara, Dalian, China), and 30.5 μl of ddH_2_O in a total reaction volume of 50 μl. The mixture was denatured at 94 °C for 1 min, followed by 33 cycles of 45 s at 94 °C, 45 s at 50 °C and 2 min at 72 °C, and a final extension at 72 °C for 10 min. There were minor modifications to the cycling conditions depending on the primers and the length of the products.

### Nucleotide sequencing and analysis

PCR products were extracted from an agarose gel using an AxyPrepTM DNA Gel Extraction Kit. Purified PCR products were then ligated into a pMD18-T vector (Takara, Dalian, China). For each product, five positive colonies were selected and sequenced. The sequence data were first checked for the quality using software Chromas, and then assembled and aligned by DNAstar software package. Phylogenetic tree based on the P1 sequences was constructed by the maximum-likelihood method and using the best model, TN93 (Tamura-Nei) with 1000 bootstrap replicates in MEGA version 5.1. The bootstrap values are shown at the branch nodes representing the most recent common ancestor of the clade they support (values< 70% not shown).

## Results

**Fig. 1:**
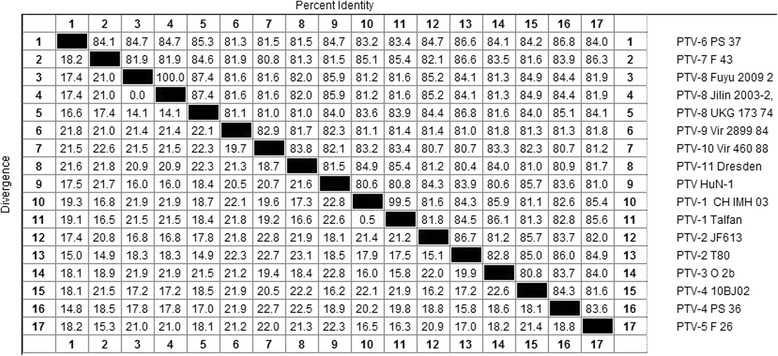
Identity of genome sequences of PTV HuN-1 isolate and other serotype reference strains

Detection of the CPE samples was PTV positive. The results showed that the PTVs could persistently proliferating in PK-15 cell. The sample of PTVs was isolated and purified by the plaque three times. Based on the published genomic sequence of PTVs, the whole genome of PTV isolate named HuN-1 was sequenced by using specific primers. The genome sequences of the PTV HuN-1 isolate were 7098 nucleotides in length, and contains one ORF (from 320 to 7039 nt) coding a 2240 amino acid polyprotein. This was preceded by a 319-nt-long 5′-UTR and followed by a 59-nt-long 3′-UTR. The coding regions contain four parts: L region(354 nt) coding L-protein; P1 regions coding structural proteins VP4(222 nt), VP2(840 nt), VP3(726 nt) and VP1(792 nt); P2 regions coding nonstructural proteins 2A(63 nt), 2B(438 nt) and 2C(963 nt); P3 regions coding nonstructural proteins 3A(273 nt), 3B(75 nt), 3C(615 nt) and 3D(1356 nt).The base composition of the full-length genome of HuN-1was found to be 27.97% A, 22.03% G, 28.18% U and 21.82% C. Sequence analysis based on the complete genomic sequences of the isolate in this study and the other known serotype reference strains, the results show that HuN-1 shares the highest nucleotide sequences identity (85.9%) with the PTV 8 strain of Jilin/2003/2(JN710381) and Fuyu/2009/2(KC757344), and as Fig. 1 showed.

**Fig. 2:**
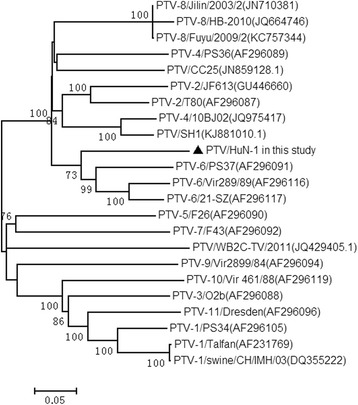
Phylogenetic analysis of PTV HuN-1 together with those of reference representative strains based on the P1 gene (2580 nt). Filled triangle HuN-1 in this study

To determine genetic relationships of PTV HuN-1 isolate with the reference strains [19], twenty two representative strains were selected to construct the phylogenetic tree (as Fig. 2 showed) on the basis of P1 sequences. The result showed that HuN-1 isolate did not cluster with the hitherto reported strains, forming a subgroup in the PTV cluster.

The genomic sequence of PTV HuN-1 isolate has been deposited in GenBank under accession number: KU297677.

## Discussion

PTVs were identified during serious outbreaks of polioencephalomyelitis in Europe in 1929 and 1957 respectively [12, 13]. PTVs were first reported in Inner Mongolia, China, 2003. Subsequently, PTVs were reported in Hebei, Beijing, Shanghai, and Heilongjiang Province [20, 21]. Now, a porcine teschovirus HuN-1 was found that it could be proliferated in PK-15 cell, and it came from the pig fecal samples from China. The present study showed that the isolation of PTV strains from domestic pigs is easily accomplished in PK-15 cell directly from pig stools.

A pair of primers (Table 1, P4-F and P4-R) were designed to amplify PTV fragments of the CPE samples for the high-mutation region VP1. The results showed that the CPE samples’ product sequences were identical. No discrepancies of sequence fragments were found in the cultures purified subsequently. Therefore, we proposed that only one teschorvirus isolate was separated from the pig feces’ sample. A pig farm may have multiple serotypes of teschorvirus, but only this virus was isolated from the experiment. To my way of thinking the reason may be that HuN-1 is a strong virulent strain, the others are weak strains, and the rapid increase of HuN-1 inhibits the increase of other strains. After many passages, HuN-1 occupies an absolute advantage, so the detected sequence is HuN-1 and no similar sequences were yielded from the isolate.

The genome of the HuN-1 isolate was amplified and sequenced, exclusive of the 5′poly(C) and the 3′poly (A) tract. The single ORF is translated into a polyprotein, which is subsequently processed. Initiation of protein synthesis on the PTV RNA could occur at one of two different AUG codons [22]. In the PTV HuN-1 sequences, there were two initiation codon, the first one at position 320, and the second one at position 416. However, comparison with other PTV sequences indicated that the first of these AUGs was not conserved, even though an extremely high level of sequence identity between different strains of PTV in coding region of leader protein. The basic organization of the product is similar in several picornaviruses. The primary cleavages separate the polyprotein into three or four regions, L, P1, P2, and P3 [3, 18]. The P1 region is precursor to the structural proteins, and P2 and P3 regions split into the nonstructural proteins responsible for replication and proliferation. The leader protein, P2, and P3 is quite conserved among the teschoviruses.

For PTVs, most genomic variations occur in coding region of the structural protein [4]. Based on the molecular comparisons presented in this study, the entire P1 region encoding structural proteins displayed high rates of mutations [18]. The complete P1 nucleotide sequences were used for phylogenetic analysis and serotype typing in this study, the result showed that this virus HuN-1 did not cluster to reported reference strains. So we speculate the HuN-1 might belongs to another new serotype, although there is still insufficient data to support this proposition. Until now, more than thirteen serotypes have been identified [5]. They were popularly mixed distribution in pig farms. In China, PTV2, PTV4, and PTV8 were the main serotypes in epidemic [23].

## Conclusions

In this study, complete genome of PTV HuN-1 was cloned and sequenced. The genome sequences of the HuN-1 isolate were 7098 nucleotides in length, and contains one ORF (from 320 to 7039 nt) coding a 2240 amino acid polyprotein. Phylogenetic analysis showed that HuN-1 did not cluster with the hitherto reported strains, forming a subgroup in the PTV cluster. Detection and characterization of further PTV strains from different geographic areas are important to understand the worldwide distribution and heterogeneity of PTVs and their association with symptomatic infections in pigs.

## Abbreviations

nt: Nucleotide
ORF: Open reading frame
PK: Porcine Kidney
RT-PCR: Reverse transcription-polymerase chain reaction
UTR: Untranslated Region
VP1: Viral protein 1
